# Apoptosis and Mobilization of Lymphocytes to Cardiac Tissue Is Associated with Myocardial Infarction in a Reperfused Porcine Model and Infarct Size in Post-PCI Patients

**DOI:** 10.1155/2018/1975167

**Published:** 2018-03-18

**Authors:** Maria J. Forteza, Isabel Trapero, Arantxa Hervas, Elena de Dios, Amparo Ruiz-Sauri, Gema Minana, Clara Bonanad, Cristina Gómez, Ricardo Oltra, Cesar Rios-Navarro, Daniel F. J. Ketelhuth, Julio Nunez, Francisco J. Chorro, Vicente Bodi

**Affiliations:** ^1^Department of Medicine, Cardiovascular Medicine Unit, Center for Molecular Medicine, Karolinska Institute, Karolinska University Hospital, Stockholm, Sweden; ^2^Department of Medicine, University of Valencia, Valencia, Spain; ^3^Institute of Health Research INCLIVA, Valencia, Spain; ^4^Department of Cardiology, Hospital Clinico Universitario de Valencia, Valencia, Spain; ^5^Department of Pathology, University of Valencia, Valencia, Spain; ^6^Intensive Care Unit, Hospital Clinico Universitario de Valencia, Valencia, Spain; ^7^Centro de Investigación Biomédica en Red-Cardiovascular (CIBERCV), Madrid, Spain

## Abstract

ST-segment elevation myocardial infarction (STEMI) is the most severe outcome of coronary artery disease. Despite rapid reperfusion of the artery, acute irrigation of the cardiac tissue is associated with increased inflammation. While innate immune response in STEMI is well described, an in-depth characterization of adaptive immune cell dynamics and their potential role remains elusive. We performed a translational study using a controlled porcine reperfusion model of STEMI and the analysis of lymphocyte subsets in 116 STEMI patients undergoing percutaneous coronary intervention (PCI). In the animal model, a sharp drop in circulating T lymphocytes occurred within the first hours after reperfusion. Notably, increased apoptosis of circulating lymphocytes and infiltration of proinflammatory Th1 lymphocytes in the heart were observed 48 h after reperfusion. Similarly, in STEMI patients, a sharp drop in circulating T lymphocyte subsets occurred within the first 24 h post-PCI. A cardiac magnetic resonance (CMR) evaluation of these patients revealed an inverse association between 24 h circulating T lymphocyte numbers and infarction size at 1-week and 6-month post-PCI. Our translational approach revealed striking changes in the circulating and tissue-infiltrating T lymphocyte repertoire in response to ischemia-reperfusion. These findings may help in developing new diagnostic and therapeutic approaches for coronary diseases.

## 1. Introduction

Coronary artery disease is the single most frequent cause of death worldwide. ST-segment elevation myocardial infarction (STEMI) is the most severe outcome of coronary disease, accounting for 12.8% of all deaths worldwide [1]. STEMI commonly occurs when thrombus formation leads to the complete occlusion of a major epicardial coronary vessel. Thus, myocardial infarction is associated with an inflammatory reaction, which is a prerequisite for healing and scar formation [2].

STEMI must be diagnosed and treated promptly, usually by means of percutaneous coronary intervention (PCI). Despite rapid reperfusion of the coronary artery, the acute irrigation of tissue has been associated with acceleration and increase in local inflammation [3]. Several mechanisms have been proposed to explain ischemia-reperfusion-induced local inflammation, including activation of complement and reactive oxygen species [4]. Hence, post–ischemic-reperfusion inflammation is characterized by the recruitment and activation of immune cells from the innate and adaptive immune systems [5, 6].

Upon activation, adaptive immune CD4^+^ T lymphocytes can develop into T-helper (Th) 1, Th2, Th17, or regulatory T cells (Tregs), depending on the set of costimulatory molecules and cytokines expressed by antigen-presenting cells. In general terms, Th1 and Th17 cells are considered proinflammatory, while Tregs have been described to maintain immune tolerance and homeostasis [7].

Previous studies from our group and others have shown an acute decrease in the blood lymphocyte count in patients with STEMI [8], which has been associated with more severe prognosis, represented by an increase in infarct size and microvascular obstruction, measured by cardiac magnetic resonance (CMR) [9]. While in general, the dynamics of innate immune cells in post–ischemic-reperfusion inflammation is well described, especially monocyte-derived cells [10]; the role of adaptive immune cells remains poorly characterized. In the present study, using a well-standardized porcine ischemia-reperfusion model and investigating STEMI patients, we show that apoptosis and tissue mobilization of lymphocytes to infarcted myocardium are associated with ischemic injury and infarct size.

## 2. Methods

### 2.1. Porcine STEMI Model and Experimental Design

Twelve juvenile domestic female pigs weighing 25–30 kg were used in the study. In short, animals were sedated using IM 8 mg/kg ketamine and 0.1 mg/kg medetodimine and anaesthetized using a 10 mg/kg/h continuous IV infusion of 2% propofol. Infarction was induced inflating a 2.5 × 10 mm angioplasty balloon in the mid left anterior descending coronary artery in anaesthetized pigs. After 90 min, the balloon was deflated, and the restoration of normal coronary flow was documented by angiography. No coronary dissection or closure was detected at reperfusion or at the 48 h angiogram. After 48 h, the pigs were anaesthetized again.

Blood samples were obtained using a multipurpose catheter placed in the coronary sinus of swine before balloon inflation, after 90 min (immediately before balloon deflation) and 2 h and 48 h after reperfusion. PBMCs were isolated and frozen following the same protocol as in patients. The hearts were then arrested with potassium chloride and removed. The left ventricle was sectioned into 5 mm thick short-axis slices and incubated with a 2% 2,3,5-triphenyltetrazolium chloride (TTZC) solution for 20 min at 37°C. Finally, sections were photographed, and the infarct size was defined as the myocardial area that failed to stain with TTZC.

The Animal Care and Use Committee of the University of Valencia approved the study, which conforms to “The Guide for the Care and Use of Laboratory Animals” published by the US National Institutes of Health (NIH Publication number 85-23, revised 1996). Further details of the experimental study are described in the supplementary material.

### 2.2. RNA Isolation and Real-Time Quantitative PCR (RT-qPCR)

Frozen myocardial tissue from the infarcted, adjacent, and remote areas of the pigs was homogenized in TRIzol isolation reagent (Life Technologies, Madrid, Spain) for RNA isolation. RT-qPCR was performed using an ABI Prism 7900 sequence detection system (Life Technologies, Madrid, Spain) with TaqMan Gene Expression Assays (Life Technologies, Madrid, Spain). The fold change in gene expression from the control group was calculated using the 2^−ΔΔCt^ method [10].

### 2.3. Immunohistochemical Characterization of Lymphocyte Infiltrates in Porcine Hearts

Tissue samples were obtained after the extraction and slicing of the heart. The samples were fixed in 10% formalin and embedded in paraffin. Afterward, 4 *μ*m thick myocardial samples from paraffin-embedded samples were histologically characterized in the infarcted, adjacent, and remote areas. The following primary antibodies were used: rabbit anti-human CD3 for T cells and mouse anti-human CD79a for B cells (both from Dako, Barcelona, Spain). Sections were then incubated with a HRP-conjugated secondary antibody and developed with 3,3′-diaminobenzidine tetrahydrochloride (Dako, Barcelona, Spain).

### 2.4. Patient Study Design and Groups

One hundred and thirty-five consecutive STEMI patients that referred to PCI during December 2011 to June 2013 were prospectively included in this study. STEMI was defined according to the latest universal definition of myocardial infarction [11]. Patients with a history of previous myocardial infarction were not considered for participation.

**Figure 1:**
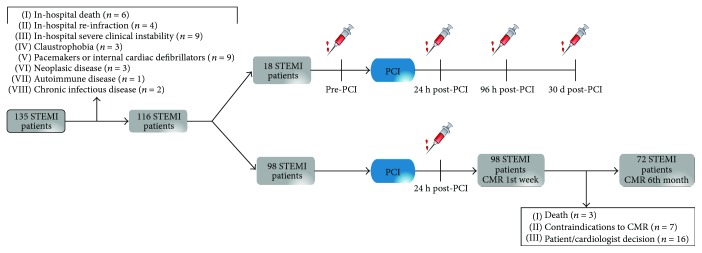
Flowchart of the study group. This flowchart illustrates the STEMI patients and controls and the different blood samples that were obtained. In total, 116 STEMI patients were selected after exclusion criteria: 18 STEMI patients were selected for serial blood extractions at different time points and 98 STEMI patients were selected for 24 h post-PCI blood extraction and CMR during 1st week and after 6 months. STEMI: ST-segment elevation myocardial infarction; CMR: cardiac magnetic resonance.

The final study group comprised 116 patients who fulfilled the inclusion criteria. From all patients, ninety-eight were assigned for blood sampling 24 h post-PCI and CMR within the first week after STEMI. Seventy-two of the ninety-eight patients repeated CMR after 6 months—twenty-six patients were excluded due to death (*n* = 3), contraindications to CMR (*n* = 7), or the cardiologist's decision (*n* = 16). Eighteen STEMI patients were assigned to serial blood sampling, including pre-PCI, and 24 h, 96 h, and 30-day post-PCI (flowchart of the overall study design is shown in Figure 1).

Individuals were managed both in-hospital and after discharge by a specific STEMI unit, and current recommendations were strictly followed. ECG and angiographic characteristics were prospectively recorded in all cases upon patient admission. Written informed consent was obtained from all patients. The study was approved by the ethical committee of clinical investigations of the Hospital Clinico Universitario de Valencia (approved in April 2008) and was conducted in agreement with the ethical principles for medical research involving humans from the Declaration of Helsinki.

The clinical characteristics of both groups are shown in Table 1. The electrocardiographic, laboratory, and angiographic characteristics of STEMI patients are shown in Supplementary Table 1. STEMI patients were examined with a 1.5-Tesla system (Sonata Magnetom, Siemens, Erlangen, Germany) in accordance with our clinic's protocol [12] (see Supplementary Materials for detailed CMR protocol).

### 2.5. Blood Collection and PBMC Isolation

Peripheral venous blood (20 ml) was drawn from all patients and controls. Total leukocyte cell count was determined using an automated blood cell counter. The peripheral blood mononuclear cells (PBMCs) were obtained using a density gradient centrifugation with Lymphoprep© (Axis-Shield, Norway). Following isolation, the PBMCs were frozen in freezing medium (10% DMSO and 90% fetal bovine serum) and stored at −80°C.

### 2.6. Flow Cytometry Analysis

Flow cytometric analysis was used to characterize lymphocyte subsets in isolated PBMCs. In brief, frozen PBMC aliquots were quickly thawed and counted with a Neubauer chamber. Discrimination between live and dead cells was carried out prior to analysis with 7-aminoactinomycin D (7-AAD; Beckman Coulter). The following conjugated human antibodies were used: PerCP-anti-CD3 and PCy5-anti-CD3 as pan T-cell marker (Beckman Coulter, CA, USA), FITC-anti-CD4 for T-helper cells, FITC-anti-CD8 for cytotoxic T cells, APC-anti-CXCR3 and PE-anti-CCR4 for Th1 and Th2 cells, PE-anti-FOXP3 for Tregs, and APC-anti-CD19 for B cells. For porcine samples, the following antibodies were used: FITC-anti-CD1 for B cells and APC-anti-FOPX3 for Tregs (Beckton Dickinson, NJ, USA). Lymphocyte apoptosis was analyzed in fresh blood samples by dual selectivity with Annexin V and 7-AAD. In brief, the lymphocyte population was gated, and apoptosis was determined as the percentage of cells positive for Annexin V but negative for 7-ADD.

Samples were analyzed using a BD FACSVerse flow cytometer (standard 2-laser configuration, BD, USA), and a minimum of 10,000 events was acquired. FlowJo 8.7 software (TriStar, Oregon, USA) was used for the analysis of all the acquired data.

### 2.7. Statistical Analysis

The Shapiro-Wilk normality test was applied to test for a normal distribution. Continuous variables were expressed as the mean ± SD, and comparisons were made using the repeated measures or ordinary one-way ANOVA with a Bonferroni post hoc test when applicable. Percentages were compared using a chi-square test and Fisher's exact test when appropriate. Statistical significance was considered for a two-tailed *p* value < 0.05. All statistical tests were performed using SPSS 19.0 (SPSS Inc., Chicago, IL, USA).

## 3. Results

### 3.1. Dynamics of Adaptive Immune Cells in Pigs Subjected to STEMI

**Figure 2:**
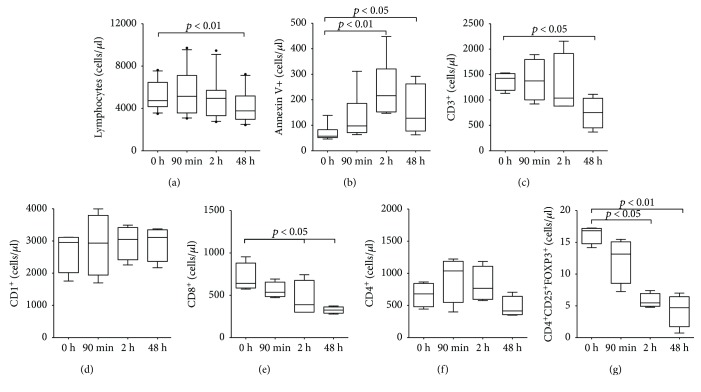
Lymphocyte and T cell dynamics in swine with induced infarction. Dynamics of lymphocyte and T cell subsets in blood from swine with induced infarction at different time points: preinfarction (0 h), postinfarction and prereperfusion (90 min), and postreperfusion (2 h and 48 h). (a) Lymphocyte cell count. (b) Lymphocyte apoptosis. Number of Annexin V-positive cells selected in the lymphocyte gate. (c) T cells (CD3). (d) B cells (CD1). (e) CD8 cells. (f) CD4 cells. (g) Treg cells (CD4^+^CD25^+^FOXP3^+^). Data are expressed as mean ± SD. versus preinfarction (0 h). SD: standard deviation; Treg: T regulatory cells.

We analyzed the dynamics of the adaptive immune cells in blood of porcine experimental model of reperfused STEMI. Corroborating with previous clinical studies from our group [13], we observed that the postischemic condition, induced by coronary occlusion and followed by reperfusion, is associated with a significant decrease circulating total lymphocyte counts (Figure 2(a)). A substantial increased apoptosis among lymphocytes was seen immediately after reperfusion and persisted over 48 h (Figure 2(b)). Among the lymphocyte subsets, we observed that T- but not B lymphocytes accounted to the drop in lymphocytes' count (Figures 2(c) and 2(d)). Interestingly, CD8^+^ T lymphocyte and Treg numbers were reduced within 2 h post-MI and stayed low till 48 h (Figures 2(e) and 2(g)). Only a modest and nonsignificant decreased CD4^+^ lymphocyte numbers were observed (Figure 2(f)).

### 3.2. T Lymphocytes Are Mobilized to Infarcted Myocardium

**Figure 3:**
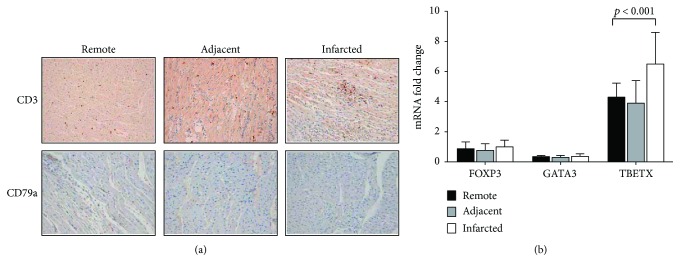
T cell infiltration and lymphocyte apoptosis in swine myocardial samples. (a) Microscopic captions of porcine myocardium; top: T cell (CD3) infiltration; bottom: B cell (CD79a) in infarcted, adjacent, and remote areas. (b) T cell subset gene expression: FOXP3, GATA 3, and TBETX in infarcted, adjacent, and remote areas. Data are expressed as mean ± SD. ^∗^ *p* < 0.05 versus remote. SD: standard deviation.

Immunohistochemical analysis of myocardium of our porcine model revealed an important infiltration of T lymphocytes to the infarcted areas of the heart (Figure 3(a), top). No change in B lymphocyte content was observed (Figure 3(a), bottom). In order to analyze the infiltrating T lymphocytes in more depth, we quantified mRNA levels of signature transcription factors for Th1, Th2, and Tregs in heart samples. Interestingly, only the transcripts for the Th1 subset, TBET, were significantly increased in the infarcted myocardium compared to adjacent and remote areas (Figure 3(b)). No changes in Th2 GATA-3 and FOXP3 Treg transcripts were observed.

### 3.3. Overview of the Dynamics of T Lymphocyte Response in STEMI Patients

Data from the porcine model suggest that lymphocytes go into apoptosis in circulation as well as Th1 lymphocytes are mobilized to the myocardium post–ischemic-reperfusion. Whether similar phenomenon occurs in humans and could influence the disease is unclear. We analyzed the dynamics of T lymphocytes in blood samples from 18 patients with STEMI, drawn at different time points—upon admission and subsequently afterwards.

**Figure 4:**
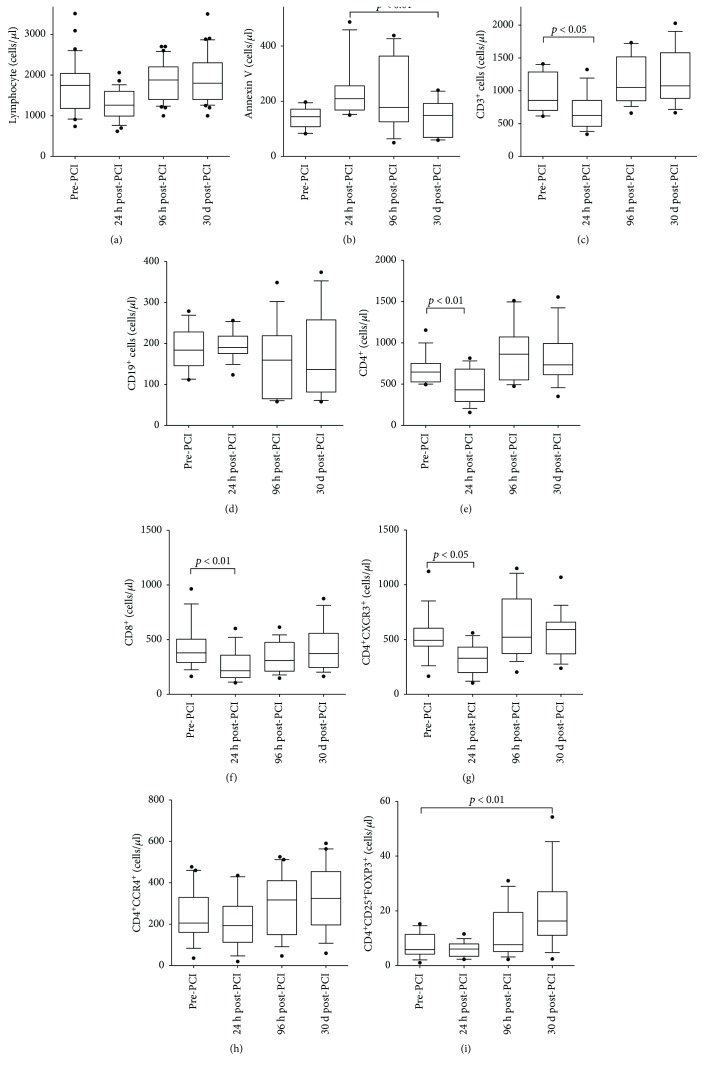
Lymphocyte and T cell dynamics STEMI patients. Total lymphocyte, lymphocyte apoptosis (Annexin V+ cells), and T cell subset count (cells/*μ*l) from blood of STEMI patients at different time points: after MI and before reperfusion (pre-PCI) and after MI and after reperfusion (24 h, 96 h, and 30 d post-PCI). A significant drop of lymphocytes, T cell (CD3^+^), CD4, CD8, and Th1 (CD4^+^CXCR3^+^) 24 h post-PCI was observed in STEMI patients. While B cells (CD19^+^) and Th2 (CD4^+^CCR4+) did not change. Treg (CD4^+^CD25^+^FOXP3^+^) increased after one month. Values are expressed as mean ± SD. *p* < 0.05 or *p* < 0.01 versus pre-PCI. PCI: percutaneous coronary intervention; SD: standard deviation; STEMI: ST-segment myocardial infarction; Treg: T regulatory cells.

As expected, lymphocyte cell counts dropped significantly 24 h post-PCI (Figure 4(a)). Lymphocyte numbers are back to baseline after 4 days. In accordance with our porcine model, lymphocyte apoptosis significantly increases post–ischemic-reperfusion and normalizes later (Figure 4(b)). Moreover, total T lymphocyte (CD3^+^) (Figure 4(c)), CD4^+^ (Figure 4(e)), and CD8^+^ T (Figure 4(f)) lymphocyte numbers dropped significantly post-PCI, whereas the number of B lymphocytes (CD19^+^) (Figure 4(d)) was unchanged. Also, in accordance with the porcine model, Th1 (CD4^+^CXCR3^+^) cells (Figure 4(g)) dropped 24 h while Th2 (CD4^+^CCR4^+^) (Figure 4(h)) cells did not change. For all the lymphocyte populations, numbers were restored to baseline 96 h post-PCI. We observed a significant increase in FOXP3^+^ Treg cells after 30 days (Figure 4(i)).

**Figure 5:**
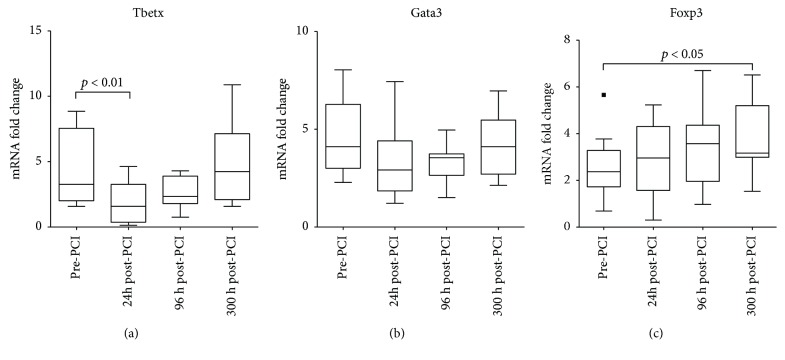
CD4 T cell subsets dynamics in STEMI patients. mRNA gene expression analysis of the dynamics of different CD4 subsets in STEMI patients. Tbetx gene expression indicates Th1 cells, Gata3 gene expression indicates Th2 cells, and FOXP3 gene expression indicates Treg cells. A significant decrease of proinflammatory Th1 cells was observed 24 h post-PCI while Treg cells increased within the next month. Values are expressed as mean ± SD. *p* < 0.05 or *p* < 0.01 versus pre-PCI. PCI: percutaneous coronary intervention; SD: standard deviation; STEMI: ST-segment myocardial infarction.

Next, we analyzed the mRNA levels of signature transcription factors for Th1, Th2, and Tregs in PBMCs of the same patients. We observed that the *TBET* transcripts were significantly reduced at 24 h post-PCI in comparison with the baseline levels (Figure 5(a)), while *GATA3* and *FOXP3* did not change at the same time point. However, increased *FOXP3* mRNA was observed 30-day post-PCI (Figures 5(b) and 5(c)).

### 3.4. Association between Infarct Size and the Dynamics of T Lymphocytes in STEMI Patients

**Figure 6:**
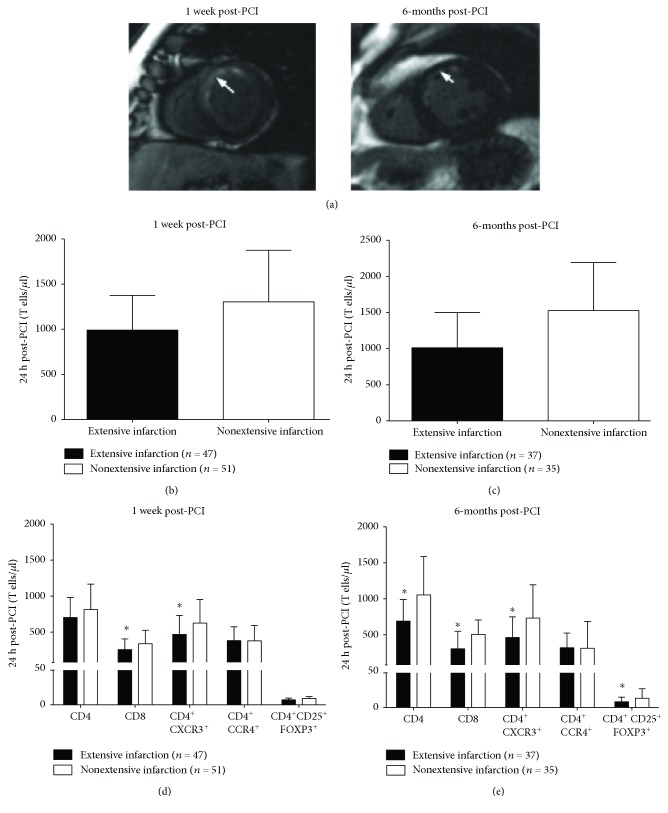
Association of CMR-derived infarct size with T cell count in STEMI patients 24 h post-PCI. (a) Example of CMR images from a patient with an anterior STEMI. (b) Association of 1-week CMR-derived infarct size with T cell count (cells/*μ*l) in blood of STEMI patients at 24 h post-PCI. (c) Association of 6-month CMR-derived infarct size with T cell count (cells/*μ*l) in blood of STEMI patients at 24 h post-PCI. (d) Association of 1-week CMR-derived infarct size with T cell subsets (cells/*μ*l) in blood of STEMI patients at 24 h post-PCI. (e) Association of 6-month CMR-derived infarct size with T cell subsets (cells/*μ*l) in blood of STEMI patients 24 h post-PCI. Data are expressed as mean ± SD. ^∗^ *p* < 0.05. PCI: percutaneous coronary intervention; SD: standard deviation; STEMI: ST-segment myocardial infarction.

Since significant changes occurred 24 h post-PCI, we aimed to investigate the association between infarct size and the T lymphocyte numbers in STEMI patients. CMR was performed in 98 STEMI patients 1 week and 6 months after PCI (Supplementary Table 2). Similar to previous studies [14], we classified patients into two groups: “extensive infarction” (IS > 18% of LV mass, median) and “nonextensive infarction” (IS ≤ 18% of LV mass) (Figure 6(a) shows representative images defining the criteria). Remarkably, patients that presented with extensive infarction at 1 week and 6-month post-PCI were those with decreased circulating T lymphocyte numbers 24 h post-PCI (Figures 6(b) and 6(c)). Moreover, extensive infarction at 1 week and 6-month post-PCI was associated with decreased numbers of CD8^+^ and Th1 subsets (Figures 6(d) and 6(e)). Extensive infarction 6-month post-PCI was also associated with lower numbers of CD4^+^ lymphocytes and Tregs at 24 h post-PCI (Figure 6(e)). No change in Th2 lymphocyte numbers was observed (Figures 6(d) and 6(e)).

## 4. Discussion

Inflammation is a very important process initiated upon myocardial injury, particularly the repair of the infarcted area. However, when out of control, this valuable mechanism can cause further damage and lead to excessive cardiac fibrosis [15, 16]. Thus, a better understanding of the cellular and molecular events associated with myocardial ischemia and reperfusion has the potential to expand and improve diagnostics and therapies for ischemic CVDs, for example, interventions that can diminish inflammatory-induced injury driven by acute reperfusion post-PCI, without interfering with myocardial healing. In the present study, using a well-standardized ischemic-reperfusion porcine model and investigating STEMI patients, we show that apoptosis and tissue mobilization of lymphocytes to infarcted myocardium are associated with the ischemic injury and infarct size.

Three main reasons led us to use a porcine STEMI model in our study: (1) a highly controlled procedure for coronary occlusion and reperfusion is in place, (2) it allowed us to obtain blood samples at crucial time points during disease development, immediately before coronary occlusion and within the subsequent 48 h, and (3) myocardial samples could be obtained to characterize tissue lymphocyte infiltration. Indeed, this systematic approach with the pigs was shown to mirror the dynamics of lymphocytes seen in STEMI patients. Notably, we confirm the previous knowledge of lymphopenia-induced postreperfusion [13, 17, 18].

The fate of lymphocytes, especially T lymphocytes, subsequent to ischemia and reperfusion has been unclear. Numerous signals derived from various stimuli, such as hypoxia-induced neoantigens, cytokines, and chemokines, have been suggested as regulators. In healthy conditions, overactivated or auto-reactive T lymphocytes are controlled peripheral tolerance mechanisms [18]. During the healing of infarcted areas of the heart, the negative regulation of the inflammatory response is critical for the protection against adverse effects that could lead to excessive remodeling and fibrosis [19].

It is well established that transient T lymphocyte depletion, largely through apoptosis, is a very important mechanism of immunoregulation [17]. This knowledge is well exemplified in the case of administration of specific T lymphocyte depleting anti-CD3 antibodies, which through the induction of apoptosis of these cells can induce a short-term immunosuppression followed by long-term tolerance [20]. In our study, ischemia-reperfusion led to a substantial increase in apoptosis of circulating lymphocytes on our pigs and patients. Although, we have not deeply investigated the molecular mechanisms that could drive this phenomenon, our data suggest that lymphopenia post–ischemic-reperfusion could be an attempt protective mechanism.

The clearance of dead cells by phagocytes has been shown to activate inhibitory programs, serving as a key mechanism for the termination of the proinflammatory cascade [20]. Along with this mechanism, increased Treg numbers may also represent an inhibitory process to stop further and unnecessary damage [21]. In line with this, increased FOXP3^+^ Treg numbers were found one month after reperfusion in STEMI patients.

Considering lymphocyte populations and subsets, the most significant changes were observed at 24 h post-PCI in patients and 48 h post coronary occlusion in the porcine model. A significant decrease in CD4^+^, Th1, and CD8^+^ lymphocytes was observed, while other subsets such as Th2 and B cells were unchanged in blood. Interestingly, analysis of pig hearts revealed an increased T lymphocyte infiltration and the expression of the signature Th1 transcription factor, TBET, in infarcted areas. Altogether, these data suggest another potential mechanism involved in the postreperfusion lymphopenia, the mobilization of cells to the myocardium.

While Th1 lymphocytes were increased in the infarcted myocardium, neither Th2 nor Treg infiltration seems to be influenced by post–ischemic-reperfusion. This pattern of cells could have direct consequences to the inflammatory process triggered in the heart. It has been shown that recombination-activating gene knockout mice (*Rag1^−/−^*), which lack T and B lymphocytes, present significantly smaller infarct size when subjected to left coronary artery ligation, compared to immunocompetent controls. Interestingly, reconstitution of *Rag1^−/−^* mice with CD4^+^ T lymphocytes from only wild type but not IFN*γ*−/− mice reversed the protective phenotype [22]. In clinical studies, it has been shown that patients with ACS present increased T lymphocyte-derived-IFN*γ* response [23, 24]. Thus, it has been recently proposed that IFN*γ* can influence TGF*β*-induced healing processes in the heart [25]. Altogether, these and our data suggest that Th1 lymphocytes and their major produced cytokine, IFN*γ*, can play a deleterious role in MI promoting cardiac damage.

The translational approach used in the present study reveals that changes in the T lymphocyte repertoire occur in a clinical scenario of STEMI patients treated with primary PCI and in a controlled experimental porcine model of induced anterior infarction. We show that the acute decrease in the proinflammatory circulating T lymphocytes in blood is due to increased apoptosis and the mobilization of these cells to the infarcted areas of the heart. Patients with extensive infarctions presented less Th1 cells in blood soon after PCI, suggesting that this early infiltration of cells could have a direct impact on myocardial inflammation and healing. These findings are very important and can help guide the development of novel diagnostic approaches and therapies for coronary diseases. Of note, in experimental models, boosting of Tregs and consequent modulation of macrophage responses have shown promising results, improving myocardium healing and increasing survival [26, 27]. The continued research in this field as well as the refinement of immunomodulatory therapies will hopefully allow us to see such strategies moving into clinical trials in the near future.

## Figures and Tables

**Table 1:** Clinical characteristics of the study and control groups.

	STEMI (*n* = 116)	Controls (*n* = 30)	*p*
Age (years)	65 ± 13	71 ± 12	n.s
Male (%)	70 (60)	19 (65)	n.s
Diabetes mellitus (%)	23 (20)	7 (23)	n.s
Hypertension (%)	79 (68)	19 (65)	n.s
Hypercholesterolemia (%)	52 (45)	17 (56)	n.s
Current smoker (%)	52 (45)	13 (43)	n.s
Previous coronary artery disease (%)	0 (0)	0 (0)	n.s

STEMI: ST-segment elevation myocardial infarction; n.s: not significant.

## Supplementary Materials

Supplementary Materials Supplementary Table 1: electrocardiographic, laboratory, and angiographic characteristics of STEMI patients. Supplementary Table 2: cardiovascular magnetic resonance imaging data of STEMI patients.
