# Supplementary material for: Apoptosis and Mobilization of Lymphocytes to Cardiac Tissue Is Associated with Myocardial Infarction in a Reperfused Porcine Model and Infarct Size in Post-PCI Patients
Source: Oxid Med Cell Longev. 2018 Mar 18;2018:1975167. doi: 10.1155/2018/1975167 (PMC5878889; doi:10.1155/2018/1975167)
Supplement: Supplementary Materials — Supplementary Table 1: electrocardiographic, laboratory, and angiographic characteristics of STEMI patients. Supplementary Table 2: cardiovascular magnetic resonance imaging data of STEMI patients. [file 1975167.f1.pdf]

Supplementary Material

Supplementary methods

CARDIAC MAGNETIC RESONANCE

Images were acquired by a phased-array body surface coil during breath-holds and were ECG-triggered. Baseline cine images were acquired in 2-, 3-, 4-chamber views and in short-axis views using a steady state free precession sequence (repetition time/echo time: 25/1.6 ms, flip angle: 61°, matrix: 256 x 256, field of view: 320 x 270 mm, slice thickness: 7 mm). Late gadolinium enhancement imaging was performed at least 10 min after administering 0.1 mmol/kg of gadoliniumdiethylenetriaminepentaacetic acid (Magnograf, Juste S.A.Q.F., Madrid, Spain) in the same locations used for baseline cine images (segmented inversion recovery steady state free precession sequence, repetition time/echo time: 750/1.26 ms, flip angle: 45°, matrix: 256 x 184, field of view: 340 x 235 mm, slice thickness: 7 mm). Inversion time was adjusted to null normal myocardium.

Left ventricular (LV) ejection fraction (LVEF, %), LV end-diastolic volume index (LVEDVI, mL/m^2), LV end-systolic volume index (LVESVI, mL/m^2) and LV mass (g/m^2) were calculated by manual planimetry of endocardial and epicardial borders in short-axis views cine images. Areas showing late gadolinium enhancement were visually revised and quantified by manual planimetry. IS (% of LV mass) was assessed as the percentage of LV mass showing late gadolinium enhancement.

CMR studies were quantified offline in a core lab (Cardiac Imaging Unit, Incliva, Valencia, Spain) by an experienced observer blinded to all patient data using customized software (QMASS MR, 6.1.5, Medis, Leiden, The Netherlands). CMR data were prospectively and immediately included into the registry database. The methodology applied for the evaluation of CMR data reproducibility can be consulted elsewhere [1]

EXPERIMENTAL STUDY

The study was approved by the Animal Care and Use Committee of the University of Valencia and it conforms with the Guide for the Care and Use of Laboratory Animals published by the US National Institutes of Health (NIH Publication No. 85–23, revised 1996). Twelve juvenile domestic female pigs weighing 25–30 kg were used. Pigs were sedated using IM 8 mg/kg ketamine and 0.1 mg/kg metodimine and anaesthetized using a 10 mg/kg/h continuous IV infusion of 2% propofol. Pigs were pre-treated with IV

amiodarone (150 mg) and lidocaine (30 mg) to reduce life-threatening arrhythmias and with heparin (3000 U). Pigs were mechanically ventilated using a 50% oxygen gas mixture. Continuous electrocardiographic monitoring of heart rate, rhythm, and ST-segment changes was performed. A 6-F sheath was introduced into the jugular vein to administer drugs and fluids if necessary.

A 6-F sheath was introduced into the right femoral artery to measure blood pressure and to access the lad left anterior descending coronary artery (LAD). A Judkins right 4 catheter and a standard hydrophilic angioplasty wire were used. Ischemia was induced by inflating a 2.5 × 10 mm balloon (Abbot Vascular, Santa Clara, CA, USA) at four atmospheres in the proximal LAD. Coronary artery occlusion was confirmed by contrast injection and by electrocardiographic ST-segment elevation. After 90 min, the LAD balloon was deflated and restoration of normal coronary flow was documented by angiography.

The animals were allowed to recover. No LAD dissection or sustained coronary closure was detected at reperfusion or at 48 h angiogram. After 48 h, 20 mL of 4% thioflavin-S solution was selectively infused into the proximal LAD using a 2.8-F microcatheter (Progreat, Terumo,

Japan). The hearts were then arrested with potassium chloride and excised.

Hearts were sectioned into 5-mm thick short-axis slices. In order to detect thioflavin-S stained areas, each slice was viewed from the apical side under ultraviolet light and photographed. Afterwards, slices were incubated in 2,3,5-triphenyltetrazolium chloride 2% solution for 20 min at 37ºC; finally, they were viewed under room light and photographed.

Images were digitalized and manual definition of endocardial and epicardial borders of all short-axis slices were carried out offline by an independent experienced investigator using the software package MATLAB 6.5 (The Mathworks, Inc., Natick, MA, USA). A ruler was photographed beside myocardial slices in all images and it was used as a reference for determining areas; this along with the pre-defined slice thickness (5 mm) permitted the calculation of volumes.

The LAD-perfused area was defined as the percentage of the myocardial volume showing thioflavin-S staining. Infarct size was designated as the percentage of the myocardial volume that failed to stain with 2,3,5-triphenyltetrazolium chloride. The salvaged myocardium was regarded as the percentage of the LAD-perfused area

showing 2,3,5-triphenyltetrazolium chloride staining. Lack of thioflavin-S staining in the core of the infarcted area was interpreted as microvascular obstruction.

RT-qPCR analysis of swine tissue

Frozen myocardial tissue from the infarcted, adjacent and remote areas of swine was homogenized in Trizol isolation reagent (Life Technologies, Madrid, Spain) for RNA isolation followed by additional column purification by RNeasy kit (QIAGEN, Madrid, Spain) and on-column DNA digestion (Life Technologies) according to manufacturer's instructions.

RNA quantity and purity were determined using the Nanodrop ND-2000 (Nanodrop, LabTech International, UK). RNA integrity was assessed using RNA 6000 Nano Labchips in an Agilent 2100 Bioanalyzer (Agilent, Santa Clara, CA, USA). RNA was reverse transcribed into cDNA with the High-Capacity RNA-to-cDNA Kit (Life Technologies, Madrid, Spain).

RT-qPCR was performed using the ABI Prism 7900 sequence detection system (Life Technologies, Madrid, Spain) using Taqman Gene Expression Assays (Life Technologies, Madrid, Spain). PCR reactions were carried out in a duplex format containing FAM-labelled (assays IDs: FOXP3 Ss03376695_u1, GATA3 Ss03388351_m1, TBETX21 Ss03373719_s1 and IL17A Ss03391803_m1) and a VIC-labelled probe as the endogenous control gene (assay ID: 18S ribosomal 4319413E).

Supplementary table 1: Electrocardiographic, laboratory and angiographic characteristics of STEMI patients

	**STEMI (n=116)**
**Age (years)**	65±13
**Male (%)**	70 (60)
**Diabetes (%)**	23 (20)
**Hypertension (%)**	79 (68)
**Hypercholesterolemia (%)**	52 (45)
**Previous coronary artery disease (%)**	0 (0)
**Current smoker (%)**	52 (45)
**Heart rate (beats per minute)**	80±17
**Systolic blood pressure (mmHg)**	126±25
**Killip class >1**	17 (15)
**ST-segment resolution (%)**	87±10
**Peak troponin value (ng/ml)**	82 [44-100]
**Time from chest pain onset to revascularization (min)**	212 [140-374]
**TIMI 3 flow pre-angioplasty (%)**	17 (15)
**TIMI 3 flow post-angioplasty (%)**	104 (90)

Abbreviations: **STEMI:** ST segment elevation myocardial infarction; **TIMI:** Thrombolysis in Myocardial Infarction

Supplementary table 2: Cardiovascular magnetic resonance imaging data of STEMI patients

	**STEMI (n=98)**
**Ejection fraction (%)**	55±12
**End-systolic volume index (ml/m^2)**	36±17
**End-diastolic volume index (ml/m^2)**	79±19
**Left ventricular mass (g/m^2)**	73±18
**Infarct size (% of left ventricular mass)**	18% [5.3-26.1]
**Patients with extensive infarction**	47

Abbreviations: **STEMI:** ST-segment elevation myocardial infarction

REFERENCES

1. Bodi V, Sanchis J, Lopez-Lereu MP, Losada A, Nunez J, et al. (2005) Usefulness of a comprehensive cardiovascular magnetic resonance imaging assessment for predicting recovery of left ventricular wall motion in the setting of myocardial stunning. J Am Coll Cardiol 46: 1747-1752.
